# Progress in Surgery

**Published:** 1900-03-31

**Authors:** 


### Progress in Surgery.

### GENITO-URINARY ORGANS.

**Exploration of the Kidney.**—Bruce Clarke,[1] in a recent address, has advocated exploration of the kidney and ureter as a conservative measure. He remarks that the assertion of some authors that if nephrotomy were more often practised nephrectomy would rarely, if ever, be needed, is probably an exaggeration. But early exploration is one of the most conservative measures in surgery; and it leaves behind a kidney as perfect in function as before, and less liable to progressive disease. The chief indication for exploration is renal pain. Formerly calculus, and calculus only, was considered the cause of renal pain. To-day calculus is looked upon as a somewhat rare cause, as compared with others. Renal distension is usually the cause of pain, just as bladder distension is usually the cause of bladder pain. Clarke relates a case illustrating this. The patient, a woman, had had the left kidney removed some years previously for calculi and suppuration. She now had pain in the right kidney, which could be felt to be enlarged, obviously hydronephrotic. The patient had learnt to manipulate the kidney, so that it emptied its contents into the bladder and relieved her of pain. She was conscious of the fact that she often had no water in her bladder for some hours, and during this time the kidney became enlarged and painful, and the above manipulations relieved this and caused an abundance of water to flow from the bladder. By operation the distended kidney was fixed to the lumbar fascia, in such a position that the water easily flowed into the bladder, and the kidney drained for a fortnight. A perfect cure resulted. Clarke quotes several other cases in which exploration of the kidney for pain paved the way to successful treatment. The cases include one of villous tumours in the kidney and one of calcareous grit in it, one of early tubercular disease, and one of peri-nephritic abscess.

Christian Fenger[2] criticises the claim recently made by Edebohls to be the originator of the plan of exploring through a lumbar incision the kidney to be left behind before performing nephrectomy. Edebohls laid down the rule that the second kidney should always be exposed and examined by the lumbar incision in contemplated nephrectomy, unless its presence and exact condition is known. Fenger claims that he advocated this proceeding before Edebohls, and details the history of a case in which he practised it in 1890. Edebohls first practised it in 1894. Rosving, a Danish surgeon, advocated the double lumbar incision in a publication in 1895.

**Tuberculosis of the Kidney.**—Watson Cheyne discussed this subject in the Harveian Lectures.[3] He remarked that the disease may be primary or secondary, that it is not uncommonly unilateral in the early stage, and that it almost always affects the ureter. Nephrotomy was the operation almost solely employed at first, the object being to establish a permanent opening in the lumbar region for the escape of pus and urine. This operation is only slightly curative. In some cases the kidney shrinks up, the disease becomes quiescent, and the fistula may be allowed to close. In the majority of cases but little benefit results from this operation. It is very difficult to prevent pus escaping down to the bladder and spreading the infection, and the opening constantly tends to contract. If it remains open the escape of urine is a great source of trouble to the patient. Nephrotomy is indicated if the patient cannot bear a severer operation, if there are peri-nephritic abscesses, and where one is not certain that the other kidney is healthy. In most cases it is to be regarded as a preliminary operation to improve the condition of the patient so that a curative nephrectomy may be performed. Before undertaking nephrectomy the condition of the other kidney and of the bladder must be ascertained. The cystoscope, and catheterisation of the ureters in females, may be used for this purpose. Abdominal exploration of the other kidney is an uncertain method, for the kidney may be tubercular, though it feels smooth and of normal size. And, again, the other kidney may be enlarged from simple hypertrophy, and the smaller kidney be the diseased one. Cheyne thinks the lumbar exploration of the other kidney the best proceeding. In some cases the matter must be left to chance. If the nephrectomy follows nephrotomy adhesions will be strong, and it may be best to remove the organ piecemeal. The whole or part of the ureter should be removed also if diseased. Some good results have been recorded of partial nephrectomy, the diseased portion being removed, and the rest left, after stitching up. Bruce Clarke[4] records a case of the kind, the affection being early. The patient was a man of 29, complaining of severe pain in the left loin and down the leg, incapacitating him. Slight attacks of hæmaturia had occurred. The lower end of the kidney on exploration was enlarged, and appeared filled with granulation tissue. It was removed, and the stump of the wound in the kidney sutured. Some urine escaped through a drainage tube for a few days. The microscope showed the growth to be tubercular. At the end of two years the patient was still well. Hiram Vineberg[5] reports a case of nephrectomy for tubercular disease, which had spread upwards to the organ from the bladder. This, he said, was a rare course for tubercular disease of the bladder to take. He did not consider it a descending tuberculosis because, when first seen, there had been absolutely no renal symptoms, and both ureteral orifices had been free. The kidney had been completely destroyed by the disease. Since its removal the patient had gained 15 pounds in weight, but the bladder symptoms remained the same. She had suffered from the latter symptoms for seven years before the nephrectomy was performed.

**Sarcoma and Carcinoma of the Kidney.**—E. Ayres[5] reports a case of sarcoma of the kidney, in which he induced labour, as there was not room for the pregnancy to go to term. Subsequently he opened the abdomen, and found the tumour to be sarcoma of the kidney. The separation of adhesions excited severe hæmorrhage, from which the patient died. The tumour had been diagnosed as sarcoma of the ovary. Robert Abby[2] has successfully removed an enormous sarcomatous kidney from an infant nine months old. The tumour sprang from the left loin, crowding the colon far to the right of the median line, and filled threequarters of the distended abdomen. The tumour had been observed at birth, and its size had increased parallel with that of the child. The operation was conducted with very careful preparations, including special warming of the room, securing the child to a heavy ironing board, previously heated, enabling the Trendelenburg position to be used, wrapping up the extremities well in cotton and flannel, and keeping the uncovered abdomen and chest constantly warmed with steaming towels. Not more than an ounce of blood was lost. The median incision above the umbilicus showed the peritoneum pushed to the right of this line. A cross cut from the navel to the lumbar region gave free access to the growth. The little patient made an uninterrupted recovery. The remaining kidney gradually increased in function until it was secreting much more than normal for both. The child quickly gained colour and flesh, and became as fine in health and activity as its sisters. The removal seemed to be clean and complete. The tumour weighed four pounds, the child fifteen before operation. Microscopic examination showed that the tumour involved the entire kidney, and was enclosed in its capsule. Portions of the cortical substance were recognisable in the periphery of the growth. There were numerous degeneration cysts present. The bulk of the tumour was a pure spindle-celled sarcoma, with no traces of muscle-fibre. It was thought probably to have arisen in the intertubular connective tissue. C. A. Morton[6] adds another to the list of cases immediately successful. The patient died five months later from tuberculosis. Morton quotes Dr. G. Walker's paper on the subject, in which that writer states the results in 145 collected cases. He found only four cases on record in which the child had lived three years after the removal of the growth, two of these being Abby's, in one of which the tumour nearly filled the abdomen. Concetti estimates that there are 7 per cent. in which there has been no recurrence for two years. Walker gives the mortality as 38·25 per cent. Morton points out that as the death-rate from the operation is 1 in 3, merely a prolongation of life for a few months resulting in those who survive, and only 4 in 145 being cured, that the parents should decide whether the risks incurred are worth the chances of benefit. His patient was 18 months old. Hæmaturia was noticed before the tumour. At the time of operation the kidney was as large as a good sized cocoanut. The microscope showed the tumour to be a round and spindle-celled sarcoma mixed with well formed gland tissue—an adeno-sarcoma.

Edebohls[7] gives in detail the history of a case of carcinoma renalis, in which he performed nephrectomy with success. Hæmaturia was first observed five months before operation. It continued a fortnight with intermittent pain in the left hypochondrium at one spot, not larger than the point of the finger, midway between the anterior superior iliac spine and the thoracic margin, and not increased by pressure. The hæmaturia, pain, and weakness, gradually became worse. A lumbar incision was made, and the kidney examined. On its anterior surface a soft, round, swelling was felt, and concluded to be a tumour. The patient was turned over, and the other kidney explored through a second lumbar incision and found healthy. The diseased kidney was then removed. On removal there was a globular tumour, two inches in diameter, on the anterior surface. Into the cavity of the pelvis protruded a small, round portion of the tumour. At one small area this was not covered by mucous membrane, and from this area probably the hæmorrhage arose. The microscope showed the tumour to be a tubular carcinoma. Eight years later the patient was well. Edebohls remarks that when a tumour becomes enlarged so as to fill the pelvis hæmorrhage ceases, as blood cannot pass down the ureter. Hæmorrhage is more likely to occur in the beginning of renal malignant tumours.

**Solitary Kidney Cyst.**—X. von Brackel[5] records the case of a young man who had for four years noticed a swelling, at first the size of a goose egg, under the ribs. The patient was liable to attacks of vomiting and constipation. At the time of operation the tumour was the size of a man's head. It was exposed extraperitoneally. The kidney was found to be healthy down to its lower pole, where the cyst was attached. Two and a half litres of bloody fluid were extracted through a cannula. The cyst was then excised, without hæmorrhage, and the kidney fixed in the loin. The kidney substance in contact with the cyst was normal. Solitary cyst of the kidney is rare, comparatively few cases having been reported, all in adults. It is generally considered a retention cyst.

[1] Clin. Jour., Jan., 1900. [2] Annals of Surgery, Sept., 1889. [3] Brit. Med. Jour., Dec., 1899. [4] Op. cit. [5] Med. Rec., Dec., 1899. [6] Brit. Med. Jour., Feb., 1900.

